# Nexus between export variety and carbon emissions in Pakistan: The role of FDI and technological development

**DOI:** 10.1371/journal.pone.0263066

**Published:** 2022-01-26

**Authors:** Ihtisham ul Haq, Bahtiyar Mehmed, Sisira Kumara Naradda Gamage, Piratdin Allayarov, Dilawar Khan, Zeeshan Zaib Khattak

**Affiliations:** 1 Department of Economics, Kohat University of Science and Technology, Kohat, Khyber Pakhtunkhwa, Pakistan; 2 Department of Economics, Neusoft Institute Guangdong, Foshan, Guangdong, China; 3 Department of Economics, Faculty of Social Science and Humanities, Rajarata University of Sri Lanka, Mihintale, Sri Lanka; 4 Department of Mathematical Methods in Economics, Faculty of Digital Economy, Tashkent State University of Economics, Tashkent, Uzbekistan; 5 Institute of Business Studies, Kohat University of Science and Technology, Kohat, Khyber Pakhtunkhwa, Pakistan; Institute for Advanced Sustainability Studies, GERMANY

## Abstract

Carbon emissions constitute a large portion of greenhouse gases that are responsible for global warming and climate change. This study examines the impact of export variety on carbon emissions along with foreign direct investment (FDI) and technological development as determinants of environmental degradation in Pakistan. Moreover, this study is conducted in the context of the environmental Kuznets curve hypothesis (EKC). This study applies dynamic ordinary least squares and error correction models for long-term and short-term estimates, respectively. The results indicate that the EKC hypothesis is valid in the long term. This implies that Pakistan’s economy reached the threshold level of income, after which an increase in income was not responsible for environmental degradation. Export variety restrains environmental degradation in the short term and is not a significant factor in the long term. Energy consumption has aggravated environmental degradation, while FDI and technological development are restraining environmental degradation. Policy measures are recommended to curb environmental degradation in Pakistan.

## Introduction

**Fig 1:**
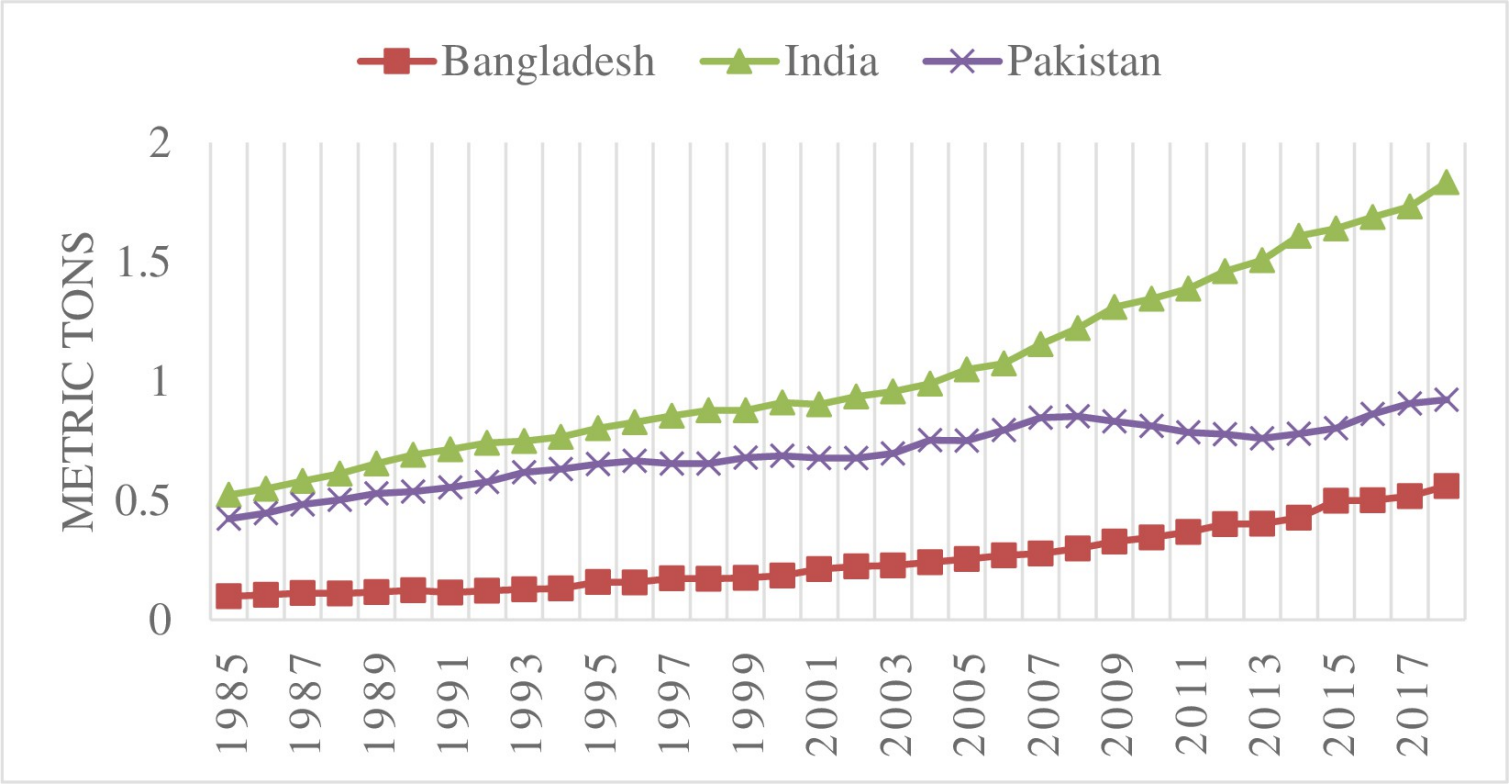
Carbon emissions per capita.

**Fig 2:**
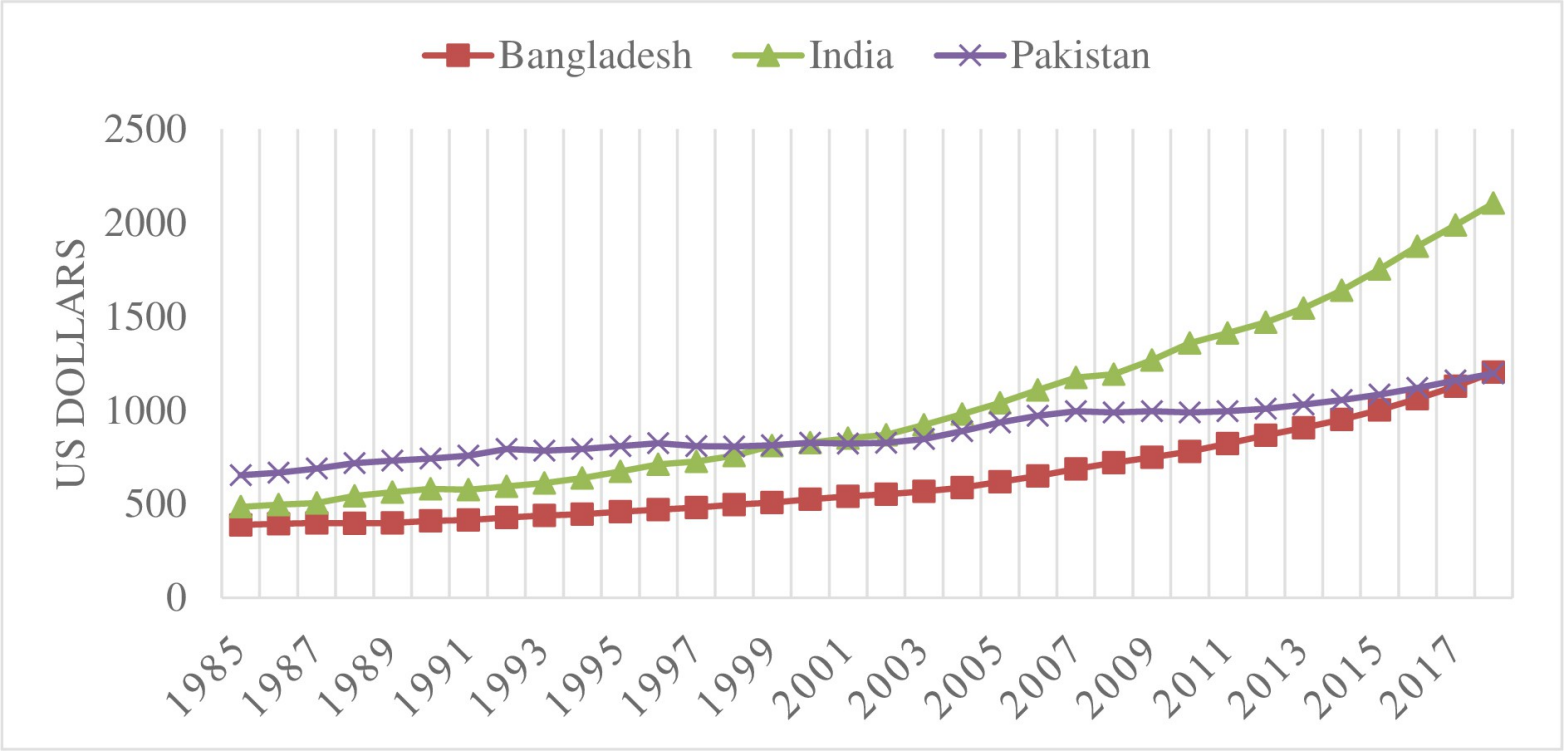
Real per capita income.

Worldwide, an enormous growth in manufacturing has occurred since the industrial revolution. Since then, there has been a constant rise in the emissions of greenhouse gases (GHGs), and approximately half of this increase has occurred in the last four decades. The period from 1983 to 2012 was the warmest thirty years in the last 1400 years. Carbon emissions from fossil fuels and industrialization have contributed a major portion of GHG emissions during the last four decades. Carbon emissions accounted for approximately 76% of GHGs, followed by methane at 16% and nitrous oxide at 6%. Since 1970, GHG emissions have increased by 75%, and energy production, agriculture, forestry and land use are the major sectors of GHG emissions [1]. GHG emissions are responsible for global warming and climate change. Pakistan has been adversely affected by climate change, and it is ranked seventh among the most climate-affected countries. Climate change is posing challenges for food and energy security in Pakistan [2]. **Figs 1 and 2** indicate carbon emissions per capita and real per capita income for Bangladesh, India and Pakistan, respectively. Between 1985 and 2018, carbon emissions per capita increased more than 5 times for Bangladesh, more than 3 times for India and more than 2 times for Pakistan [3]. This scenario of a smaller increase in carbon emissions in Pakistan compared to Bangladesh and India can be understood easily when one compares real per capita income between these countries. During the same period, Bangladesh and India had increases of more than 3 times and 4 times in real per capita income, respectively, whereas Pakistan had an increase of less than two times [4].

Trade liberalization leads to more foreign trade, and the trade structure (composition) changes with the passage of time, as not only more goods but also a greater variety of goods are traded. However, emissions embodied in trade are responsible for environmental degradation and global warming [1]. Thus, trade as a determinant of environmental degradation has received attention in empirical studies, although the impact of trade on environmental degradation has not been settled in the literature. Studies such as [5] concluded that trade deteriorates environmental quality, whereas [6] concluded that trade improves environmental quality. Both of these studies argued that trade structure matters for environmental degradation. Moreover, [7] argued that it is important for policy makers to know the individual effects of exports and imports on environmental degradation. As for as, exports are concerned, it makes a country to integrate with rest of world and consequently the export sector may expose to strict environmental standards that may be imposed by leading import partners. Thus; the strict environmental standards encourage the exporter country to adopt cleaner production techniques and technologies [8]. This is the reason that many researchers have determined the effect of exports on environmental degradation [8–12]. Several researchers have included export diversification as a determinant of environmental degradation [13–15], whereas the study conducted by [16] investigated the effect of export variety on environmental degradation.

Foreign direct investment (FDI) can lead to economic growth, as it helps in the transfer of technology from developed countries to developing countries. However, FDI-induced economic growth may not be without environmental costs [17], and hence, FDI also receives consideration from researchers as a potential driver of environmental quality in the host country. Comparatively, in developing countries, FDI can add to dirty industries more than in developing countries, where they have more concern about economic growth and where it is generally believed that developing countries may undervalue the environment to attract more investment [18, 19]. The effect of FDI on the environment can be decomposed into scale and composite effects. The FDI scale effect refers to FDI-led growth, as FDI depletes resources to boost output, thus depleting environmental quality. The composite effect of FDI refers to a decrease in price due to economies of scale, and the positive effect of FDI on income will arouse concern about environmental quality. When FDI acts to improve the environment in the host country, it is mentioned as a halo hypothesis in the literature [20, 21].

In his study, [22] argued that enhancing production, especially in the industrial sector, would lead to a rise in environmental degradation. However, [23] concluded that the use of improved technology and investment in green technology will not only improve the per capita income but also ensure fewer carbon dioxide emissions. Similarly, a number of studies [24–26] emphasized technological development for better environmental quality, as these studies concluded that improved technology would help countries emit less carbon dioxide. However, it is not always the case that technological improvement will reduce environmental degradation. As pointed out by [27], it is not technological innovations that necessarily reduce environmental degradation; instead, environmental quality is improved by pollution control policies.

In a study [5] for Pakistan, researchers argued that imbalanced economic development of Pakistan is responsible for environmental issues, for instance, shifts from agricultural and conventional production to modern industrial production techniques made Pakistan economically progressive but at the expense of environment. Likewise, industrialization in Pakistan leads to high emissions and consequently, deteriorated environmental quality. On other hand, high economic growth on average from 1970s to 2008 resulted in high energy demand that changed the composition of energy supply in Pakistan and the share of thermal energy increased especially in electricity production. Henceforth, accumulation of carbon emissions aggravated in Pakistan [5]. Besides, composition of Pakistan’s economy portraits that the share of agriculture which was around 32% in GDP during 1961–1984 has been dropped to 23% during 1985–2018 while the share of industry remains around 20% and annual growth in industrialization is above 6.5% during 1961–2018. The employment scenario also changed as the share of agriculture dropped from 45% to 37% during 1992–2018 while share of industry in total employment increased from 21% to almost 25% during 1992–2018 [4]. These arguments provide good background for commencement of this study.

The aim of the current study is to determine the effect of export variety on carbon emissions by considering the role of FDI and technological development in Pakistan. This study is the first to examine the effect of export variety on carbon emissions, as we did not find any study for Pakistan on this topic. Although [25] determined the effect of technological development on environmental degradation for Pakistan, this study is different from his study on the basis of two distinct points. First, this study is carried out in the conventional environmental Kuznets curve (EKC) hypothesis framework by considering per capita income and its square, whereas [25] was not done in EKC hypothesis framework. Besides, [25] considered economic growth, and tourism development along with technological development as determinants of environmental degradation. Second, the study conducted by [25] used patent applications as a proxy for technological development, whereas this study uses the KOF informational globalization index as a proxy for technological development because this index is composed of patent applications, internet usage and high-tech exports. Therefore, we believe that the KOF informational index is a more comprehensive index than patent applications in terms of technological development. This study also differs from the study conducted by [17], which determined the effect of export variety and FDI on carbon emissions in India, for two reasons. First, their study was not carried out under the conventional EKC hypothesis. They tested the EKC hypothesis on the basis of long- and short-term estimates following [28]. Second, [17] did not consider technological development as a determinant of carbon emissions, whereas this study also accounts for the technological development and carbon emissions nexus in Pakistan.

## Review of literature

Industrialization is responsible for global warming and climate change. It leads not only to the depletion of natural resources but also to air, water and soil pollution. On the other hand, industrialization is one of the keys to the economic prosperity and economic development of a country, as it creates job opportunities and leads to wealth generation. However, in the early stage of economic development, people do not care for the environment, and an increasing number of natural resources are depleted as they strive for basic necessities and livelihoods. However, after reaching a certain stage of development, as income increases, people start to think about the environment, and after a certain threshold income level, a further increase in income level decreases environmental degradation. This phenomenon is known as the environmental Kuznets curve (EKC) hypothesis in the literature [29]. To test the EKC hypothesis, a number of empirical studies incorporated the square of income per capita along with income per capita to affirm the EKC hypothesis [12, 30–32]. In the same manner, one can trace many empirical studies that did not confirm the EKC hypothesis [33–35].

The production and consumption of energy is a major element of carbon emissions; thus, researchers have analyzed energy as a potential determinant of environmental degradation. Energy is an essential factor of any economic activity, and countries usually achieve high economic growth where energy consumption is high; however, carbon emissions deplete environmental quality. Studies that included energy consumption as a factor of environmental quality include [5, 6, 12, 30, 31, 35, 36], among others. The impact of energy on environmental degradation depends on the energy mix of a country, and this may improve or degrade environmental quality.

Trade as a potential determinant of environmental degradation has received attention from researchers. [7] opined that it is advised to determine the individual impact of exports and imports on environmental degradation from a policy perspective. However, more emphasis is given to exports than imports. Why is it crucial to examine the nexus between exports and carbon dioxide emissions? There are certain reasons for this. One is that it is important to examine this relationship because of the market response to exports. A rise in exports leads to more demand for manufactured goods, and as a consequence, this leads to more demand for energy consumption, thus resulting in more carbon dioxide emissions and additional pressure on environmental quality. Another possible reason is that the relation between exports and carbon dioxide emissions facilitates evaluation of the careful use of resources if exports are inducing carbon dioxide emissions and responsible for an increase in demand for energy. Countries are required to determine suitable channels of fossil fuels to alleviate the complete depletion of resources. This argument means that countries have to consider more production to meet the demand for exports and have to care about the rise in carbon dioxide emissions. Moreover, countries have to consider export and carbon dioxide emissions relationships without exhausting energy resources and have to carry out energy production processes that generate more energy and allow fewer emissions into the environment [37].

[14] analyzed the impact of export diversification on environmental degradation in China, Korea and Japan. The results of their study documented that export diversification leads to environmental degradation. Likewise, [15] carried out a study for developing countries to examine the effect of export diversification on environmental degradation. They concluded that export diversification positively contributes to carbon emissions. Recently, [38] examined the effect of exports and imports on environmental degradation in Ghana. The study concluded that exports and imports have insignificant effects on environmental degradation in the long run, but exports have significant negative effects on environmental degradation in the short term.

Sustainable development refers to fulfilling the present needs of society without compromising the ability of future generations to fulfill their own needs, and sustainable manufacturing is one of the ways to lessen the environmental impacts of products [39]. The impact assessment of products is described at the endpoint level as well as the midpoint level. The former impacts damage health, biodiversity or ecosystems, whereas the latter impacts resource depletion, acidification and global warming [40]. Consumer needs vary with situations, constraints, and social values; thus, products are designed and manufactured to fulfill these perceived needs. Producers offer diversified products to meet consumer needs. Producers believe that they can not only evade competition by offering a greater range of products but also enhance profit by increasing the variety of the product line. Product variety (PV) can be defined as an instance of a product class that possesses slight differences from the common type of the product. PV can also be defined as a range of different characteristics of one particular product class [41]. Moreover, consumers love variety, and PV is theoretically subject to supply-side factors. Any variation in supply factors will determine the level of PV, even though the demand for PV is unlimited. However, if one limits the demand for variety by taking into consideration environmental quality over the excessive range of variety, consumers might be conscious about the pollution produced due to the greater availability of PV. Thus, consumers’ love of variety could influence demand and the optimal level of PV because of its effect on environmental degradation [42]. This argument provides the basis for export variety as a determinant of environmental degradation. Therefore, [16] examined the effect of export variety on environmental degradation in India. They concluded that enhancing export variety would deteriorate environmental quality, as it would lead to more carbon emissions.

The effect of FDI on environmental degradation has not been settled in the literature. FDI may improve environmental quality or may deteriorate it. A number of studies have investigated the effect of FDI on environmental degradation [43, 44]. Many of the studies confirmed the pollution haven hypothesis about FDI and concluded that FDI deteriorates environmental quality [43, 45–47]. However, other studies confirmed the halo hypothesis about FDI and concluded that FDI improves environmental quality [48–50]. In a study, [51] determined the effect of FDI on carbon emissions in the Laos economy. They applied unit root tests for the nonstationarity problem, the Johansen cointegration test for the long-term relationship and an error correction model for the short-term dynamic effect of FDI on carbon emissions. The results of that study indicated a long-term relationship between the studied variables. Moreover, the study concluded that an inverted U-shaped relationship exists between FDI and carbon emissions. In addition, the economic growth and industrial structure of Laos are also responsible for environmental degradation.

Technological development has a great importance not only for environmental issues but also for climate change. Its importance can be gauged from its effects on the energy mix and energy efficiency from greener and more efficient technologies [52]. Recent studies have investigated the effect of technological development on carbon emissions [53, 54]. All of these studies concluded that technological development is helpful to mitigate carbon emissions. In a recent study, [25] examined the effect of technological innovation on carbon emissions in Pakistan. The results showed that economic growth and tourism development are positively associated with carbon emissions, whereas technological innovation is negatively associated with carbon emissions in both the long and short term. They argued that technological innovation would bring efficiency not only in production but also in the use of energy resources; thus, technological innovation could improve environmental quality.

It can be concluded from the above literature summary that export variety, FDI and technological development can be considered determinants of carbon emissions in the conventional EKC hypothesis setting. Hence, the empirical model of the study is developed and expressed as follows:

$$\log\;CEP_{t}=\alpha_{0}+\beta_{1}\log PC_{t}+\beta_{2}\log PC_{t}^{2}+\beta_{3}\log EC_{t}+\beta_{4}\log XPV_{t}+\beta_{5}\log FDI_{t}+\beta_{5}\log TD_{t}+e_{t}$$ (1)

where *CEP*, *PC*, *PC*^2^, *EC*, *XPV*, *FDI*, and *TD* denote total carbon emissions per capita, income per capita, income per capita square, energy consumption per capita, export variety, foreign direct investment and technological development, respectively; *β*_*i*_ are the coefficients of the variables; *t* denotes the time period; *e*_*t*_ is the residual term; and *log* is the natural log of the variable. If *β*_1_ = *β*_2_ = 0, there is no relationship between environmental degradation and per capita income. If *β*_1_>0 and *β*_2_ = 0, there will be a monotonically increasing relationship between environmental degradation and per capita income such that economic growth will be followed by a rise in environmental degradation. If *β*_1_<0 and β_2_ = 0, there will be a monotonically decreasing relationship between environmental degradation and per capita income. If *β*_1_>0 and *β*_2_<0, a classic inverted U-shaped relationship exists between environmental degradation and per capita income, and the EKC hypothesis will be tested. If *β*_1_<0 and *β*_2_>0, a U-shaped relationship exists between environmental degradation and per capita income. *β*_4_, *β*_5_, and *β*_6_ may be >0 or <0. Additionally, two advantages are associated with the log-linear model. First, one can interpret coefficients as elasticity of the respective variable. Second, it helps to address the heteroskedasticity problem as variables are transformed into natural logs.

## Research methodology

In this study, carbon dioxide emissions are used as a proxy for environmental degradation, as this is commonly used in related research [6, 55]. Moreover, another reason for the use of carbon dioxide emissions is that they constitute more than 80% of the total global greenhouse gas emissions. In addition, carbon dioxide emissions as a proxy for environmental degradation leads to a production-based approach in the context of the EKC hypothesis, as this variable does not take into account emissions from imports. Data on carbon dioxide emissions are collected from British Petroleum [3] and are divided by the total population to adjust for the impact of population growth on the carbon dioxide level. It is measured in metric tons per capita. Gross domestic product per capita is used as a proxy for income per capita and is in constant US dollars. The source for income per capita and FDI inflows is the world development indicators [4]. Energy consumption is indicated by primary energy consumption per capita, in which total primary energy consumption is divided by population. It is collected from British Petroleum [3]. Export variety is determined through the entropy index following [56], who determined export variety through this index in their study of Pakistan. Thus, to measure export variety in Pakistan, disaggregated export data at the five-digit SITC level are collected from the United Nations commodity trade statistics [57], and NACE revision classifications are used to categorize data into industrial sectors. NACE is an acronym used to designate the classification of economic activities by the European Union. [58] pointed out that the entropy index can be used to measure export variety. The coefficient of entropy (*H*) describes the expected informational content or uncertainty of a probability distribution. This index is shown in Eq 2 below:

$$XPV=H=\sum_{i=1}^{n}p_{i}\log_{2}(\frac{1}{p_{i}})$$ (2)

where *n* represents the number of sectors in the economy and *p*_*i*_ shows the share of each sector in exports. This index can be decomposed at each sector level without the problem of collinearity [59]. This index takes its minimum value (0) if exports are concentrated in one sector (*p*_*i*_ = 1 *for i* = 1; *p*_*i*_ = 0 *for i* = 2,…,*n*) and takes its highest value if exports are highly diversified, corresponding to the situation of equal shares ($p_{i}=\frac{1}{n},\forall p_{i}$).

As for a proxy for technological development, most research studies have used patent applications [53, 55]. In these studies, researchers collected data on the number of patent applications that residents and nonresidents applied for and then aggregated them to capture the total effects of technological development on environmental degradation. However, in this study, we use the KOF informational globalization index as a proxy for technological development, as this index is composed of patent applications (residents plus nonresidents), internet usage and high-tech exports. Therefore, we believe that the KOF informational globalization index is a more comprehensive index compared to patent applications for technological development. Moreover, the concept of including technological development as a determinant of environmental degradation is that countries should invest in technological development, as countries can reduce environmental degradation through improvement in technologies, especially by adopting and promoting energy technologies with lower emissions. In addition, countries should encourage the efficient use of energy along with the use of alternative energy resources [60, 61]. This study covers the period from 1985 to 2018 based on the availability of data.

It is recommended to test nonstationarity for time series analysis, as most time series data have temporal trends and do not have a constant mean or variance. If data do not possess such properties, one cannot apply ordinary least squares, as this can lead to spurious regression, which produces unreliable results. To check variables for the nonstationarity problem in time series, two distinct unit root tests of [62, 63] are applied. After confirming the order of integration of the time series, this study will apply Johansen and Juselius cointegration to determine long-term associations among variables [64]. The main benefit of this test is that it takes the variables on first difference and has the ability to not lose any long-term information that the data may possess. Once cointegration is completed, long-term estimates will be obtained through dynamic ordinary least squares (DOLS) cointegration regression as in [65]. DOLS performs well in determining the long-term relation between exploratory and explanatory variables, as it takes leads and lags of explanatory variables in the process of cointegration regression. In addition, it alleviates the feedback effect that may arise during the cointegration process. Moreover, long-term estimates obtained through DOLS are efficient, as this approach addresses autocorrelation and simultaneity bias. Finally, this study applies an error correction model based on cointegration to obtain short-term results.

## Interpretation of results and discussion

The results of the PP and ADF unit root tests are presented in Tables 1 and 2, respectively. The results of both unit root tests indicate that the variables of the study suffer from nonstationarity when in their original form. However, when variables are taken at their first difference, the problem of nonstationarity is eliminated; thus, the order of integration of the study variables is concluded to be one. These results of the unit root tests indicate that one can apply the Johansen cointegration test, as all variables of the study are integrated at the same order. Table 3 presents the long-term relationship results obtained through the cointegration test. The trace statistic and maximum eigenvalue statistics of the cointegration test confirm two cointegration vectors. Thus, the null hypothesis of no cointegration is rejected because there are two cointegration vectors, meaning that there is a long-term relationship between the variables of the study. Therefore, one can apply cointegration regressions to obtain long-term results.

**Table 1:** PP unit root test results.

Variable	PP adj. t-stat.	Variable	PP adj. t-stat.
*logCEP*	-2.31	*ΔlogCEP*	-3.42**
*logPC*	-0.07	*ΔlogPC*	-3.27**
*logPC* ^*2*^	-0.07	*ΔlogPC* ^*2*^	-3.22**
*logEC*	-1.57	*ΔlogPEC*	-4.33***
*logXPV*	0.48	*ΔlogXPV*	-4.87***
*logFDI*	-1.51	*ΔlogFDI*	-4.65***
*logTD*	-1.34	*ΔlogTD*	-4.64***

*** and ** indicate significance at the 1% and 5% levels, respectively.

**Table 2:** ADF unit root test results.

Variable	ADF t-stat.	Variable	ADF t-stat.
*logCEP*	-1.75	*ΔlogCEP*	-3.45**
*logPC*	-0.04	*ΔlogPC*	-2.98**
*logPC* ^*2*^	-0.07	*ΔlogPC* ^*2*^	-3.20**
*logEC*	-1.68	*ΔlogPEC*	-4.27***
*logXPV*	0.38	*ΔlogXPV*	-3.39**
*logFDI*	-1.44	*ΔlogFDI*	-4.61***
*logTD*	-1.24	*ΔlogTD*	-3.88**

*** and ** indicate significance at the 1% and 5% levels, respectively.

**Table 3:** Results of cointegration test.

Null hypothesis	Trace Statistics	Critical Value	Max-Eigen Statistics	Critical Value
*r* = 0	174.52***	125.62	67.94***	46.23
*r*≤1	106.58***	95.75	40.82**	40.08
*r*≤2	65.77	69.82	25.45	33.88
*r*≤3	40.32	47.86	19.11	27.58
*r*≤4	21.21	29.80	12.37	21.13
*r*≤5	8.84	15.50	8.73	14.26
*r*≤6	0.12	3.84	0.12	3.84

*** and ** indicate significance at the 1% and 5% levels, respectively.

The long-term estimates obtained through DOLS are provided in Table 4. These results indicate that all explanatory variables are significant factors of environmental degradation in Pakistan over the long term and that the EKC hypothesis exists in Pakistan in the long term because the coefficient of per capita income is positive and significant, while the coefficient of its square is significantly negative. This means that the initial increase in per capita income of Pakistan is accompanied by environmental degradation, but after per capita income reaches a certain threshold, a further increase does not increase environmental degradation. After this threshold income is reached, a 1% increase in per capita income will decrease carbon dioxide by 0.76%. The highest real income per capita in the studied period (1985–2018) is $1196 USD, whereas the turning point based on the EKC hypothesis is $1053.63 USD. The Study [5] also confirmed the EKC hypothesis for carbon emissions in case of Pakistan however; the turning point determined for the EKC hypothesis was $ 625 USD whereas the highest real income per capita was $ 652 USD in data analyzed by [5].

**Table 4:** Long-term results of cointegration regression.

*Regressors*	*Coefficient*	*t-Statistic*	*Prob*.
*Constant*	-38.87*	-2.31	<0.10
*logPC*	10.58*	2.15	<0.10
*logPC* ^*2*^	-0.76*	-2.12	<0.10
*logEC*	1.59***	7.22	<0.01
*logXPV*	-0.11	-0.29	>0.10
*logFDI*	-0.09**	-3.45	<0.05
*logTD*	-0.12**	-2.79	<0.05

***, ** and * indicate significance at the 1%, 5% and 10% levels, respectively.

Energy consumption has a significant positive effect on environmental degradation, which demonstrates that energy consumption not only lessens environmental quality through economic activity, as pointed out by [5], but also directly contributes to environmental degradation. Why would energy not be aggravating the environmental degradation, as the share of fossil fuel is almost 60% of the total energy? Fossil fuel consumption contributes to carbon emissions; moreover, its waste is harmful to health and the environment. The comparison of emissions from fossil fuels and renewable energy resources will provide a better picture to understand how much fossil fuel consumption is hazardous to the environment. For example, coal combustion in electricity generation releases between 1.4 and 3.6 pounds of CO2E/kWh compared to hydroelectricity and wind energy, which each emit between 0.1 and 0.5 pounds of CO2E/kWh. The share of wind energy in total energy is negligible, and the share of hydroelectricity has been decreasing in Pakistan over the last couple of decades. In contrast, the share of thermal energy in total energy has been on the rise since the early 2000s as the government wants to mitigate the energy crisis in Pakistan. However, such government policies will further worsen the environmental quality in Pakistan. If we look into the energy composition in Pakistan, electricity production from renewable resources is just 0.38% if we exclude hydroelectricity production. Even recent figures also support our result in the sense that during fiscal year 2017, the share of thermal production was 64%, and the share of hydro production was reduced to 30% from 34% [1]. This is the reason that [59, 60] recommended that countries should encourage the efficient use of energy along with the use of alternative energy resources [59, 60].

The results of this study showed that the effect of export variety on environmental degradation is not significant in the long run. However, it has a negative coefficient, which highlights that producers enhancing export variety does not aggravate environmental degradation because an increase in export variety will not increase carbon dioxide emissions in Pakistan. This finding is opposed to the finding of [17], which determined a positive effect of export variety on carbon dioxide for India. In addition, [12] concluded that exports had a negative impact on carbon emissions and argued that this impact can be referred to as a composite effect of exports. The composite effect of exports prevails if exports are produced with efficient technology such that exports will not put pressure on environmental quality while being competitive in the international market. Similarly, [5] argued that the technique effect of trade might restrain environmental degradation if producers are adopting environmentally friendly technology in production. During the study period, Pakistan experienced a more than 8-fold increase in goods exports, and this study determined that export variety for goods still did not aggravate environmental degradation in Pakistan. Thus, the technique effect dominates the scale effect of exports in Pakistan, as the scale effect refers to the expansion of production and leads to more emissions. Moreover, one can conclude that producers are taking care of international standards and producing goods efficiently. Besides, goods exports to high-income economies constitute more than 60% of total goods exports. These facts about Pakistan’s exports support the argument of [8] that strict environmental standards set by leading importers standards encourage the exporter country to adopt cleaner production techniques and technologies.

Long-term estimates confirmed that FDI has a significant negative association with environmental degradation. This finding navigates the pollution haven hypothesis and supports the halo hypothesis in case of Pakistan. This finding confirms the argument put forward in studies [48–50]. Moreover, this finding also support the argument of study [51] conducted in context of Laos that the FDI level has reached to a certain level after that it improves environmental quality. Our result about FDI is inconsistent with the finding of [66] as FDI positively contributing to environmental degradation as this study confirmed positive association of FDI with carbon emissions in developing countries, Kenya and Zimbabwe. Additionally, our result also is inconsistent with the findings of [16] who finds that FDI deteriorates environmental degradation in India. The FDI coefficient is -0.09, so its magnitude is small. The reason is that the ratio of FDI to GDP has varied between 0.01% and 3% over the last three decades. However, this negative association of FDI with carbon emissions highlights that Pakistan can cope with environmental degradation by encouraging FDI.

The long-term estimates show that technological development has a significant and negative effect on carbon dioxide. This result can be interpreted as a 1% increase in technological development reducing carbon dioxide emissions by 0.12%. This finding resembles the findings of other related studies [24–26]. Moreover, this finding supports the argument of [23] that improvement and investment in technology will help reduce the emission of carbon dioxide; hence, technological development will ease pressure on environmental degradation. Moreover, this finding supports the arguments of [59, 60] that countries should invest in technological development, as countries can reduce environmental degradation through improvement in technologies, especially by adopting and promoting energy technologies with lower emissions.

Although the estimates of DOLS are efficient as it addresses the endogeneity problem however; one needs a technique that also addresses multicollinearity problem. So, for robustness check, this study applied Liu’s estimator [67]. These results are depicted in Table 5. It is worth mentioning the results of long run estimates obtained through DOLS is also confirmed by Liu’s estimator however; magnitudes are slightly different compare to results based on DOLS.

**Table 5:** Long-term results of Liu’s estimator.

*Regressors*	*Coefficient*	*t-Statistic*	*Prob*.
*Constant*	-25.77	-1.23	>0.10
*logPC*	6.54**	2.35	<0.05
*logPC* ^*2*^	-0.45**	-2.24	<0.05
*logEC*	0.75***	5.37	<0.01
*logXPV*	-0.43	-1.59	>0.10
*logFDI*	-0.06*	-2.19	<0.10
*logTD*	-0.11*	-3.77	<0.01

***, ** and * indicate significance at the 1%, 5% and 10% levels, respectively.

**Fig 3:**
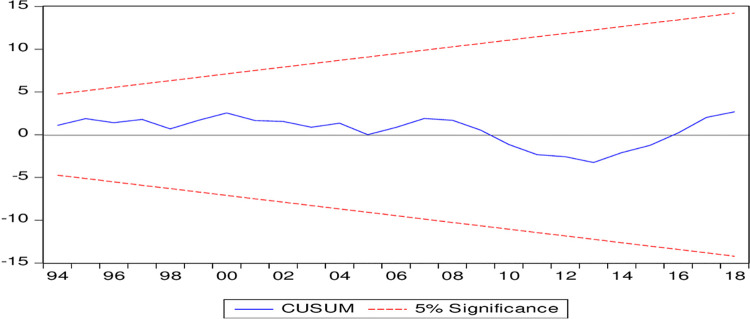
Plot of cumulative sum of recursive residual.

**Fig 4:**
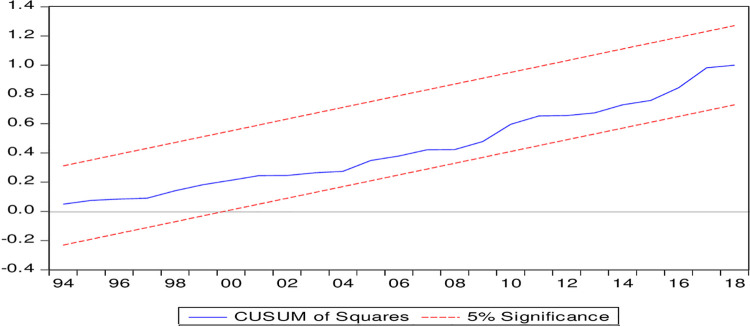
Plot of cumulative sum of squares of recursive residual.

Table 6 presents the short-term results based on the error correction model (ECM). Income per capita, its square, FDI and technological development do not have a significant effect on environmental degradation. Thus, no support of the EKC hypothesis is found in the short term. Energy consumption and export variety have positive and negative effects on environmental degradation, respectively, in the short term. Thus, energy consumption aggravates environmental degradation not only in the long run but also in the short term. Export variety restrains environmental degradation in the short term. This finding supports our argument that the technique effect dominates the scale effect in Pakistan. The model will be in equilibrium if the error correction term (ECT) of the ECM carries a negative sign and is significant. These results show that the error correction term meets those criteria; thus, it is concluded that the model is in equilibrium and will adjust itself from any external shock within two years, as the coefficient of the error correction term is approximately 0.51. The model satisfied diagnostic tests such as serial correlation, heteroskedasticity, Ramsey RESET and the Jarque-Berra normality test. Thus, the model is a free problem of serial correlation and heteroskedasticity, and the functional form of the model is correct, as suggested by the Ramsey RESET test results. Moreover, this model passed the stability test of CUSUM and CUSUM squares, as the lines of these tests are within critical boundaries. These stability test results are depicted in Figs 3 and 4.

**Table 6:** Short-term results based on ECM.

Dependent variable: Δ*log(CEP)*			
Regressor	Coefficient	t-Statistic	*Prob*.
*Constant*	0.01	1.06	>0.10
Δ*logPC*	0.88	0.19	>0.10
Δ*logPC*^*2*^	-0.07	-0.21	>0.10
Δ*logEC*	0.86***	7.77	<0.01
Δ*logXPV*	-0.53^(*)^	-1.71	<0.11
Δ*logFDI*	0.01	0.78	>0.10
Δ*logTD*	0.06	1.39	>0.10
ECT(-1)	-0.51	-3.44	<0.05
R^2^	0.81		
Adjusted R^2^	0.76		
Diagnostic Tests			
Serial correlation	0.06 (0.35)		
Heteroskedasticity	1.01 (0.44)		
Ramsey RESET	0.01 (0.81)		
Jarque-Berra	0.90 (0.64)		

*** and ^(*)^ indicate significance at the 1% and 11% levels, respectively.

## Conclusion and policy implications

The purpose of this study was to examine the effect of export variety on environmental degradation considering the role of FDI and technological development. Time series data were analyzed covering the period from 1985 to 2018. A unit root test was applied to check the nonstationarity of the time series data, and a cointegration test was applied to determine the long-term relationship between environmental degradation, income per capita, income per capita squared, energy, export variety, FDI, and technological development. The variables are free from nonstationarity at the first difference, and the results of the Johansen cointegration test confirm a long-term relationship between the studied variables. The results of the study showed that income, energy consumption, export variety, FDI and technological development are significant factors of environmental degradation in Pakistan. Moreover, the long-term results indicate that income and energy consumption positively contribute to environmental degradation, and the EKC hypothesis exists over the long run. Thus, Pakistan’s economy surpassed the threshold income level after which an increase in income was not accompanied by environmental degradation. This study finds that export variety is insignificant in the long term but carries a negative sign, whereas FDI and technological development restrain environmental degradation because they have a negative impact on environmental degradation in Pakistan over the long run. Short-term results show that energy is also responsible for environmental degradation, whereas export variety restrains environmental degradation. Moreover, the model of the study is dynamically stable and will adjust itself from any external shock by 51% annually. Thus, the model will return to equilibrium within two years from any disequilibria due to external shocks.

During the studied period, Pakistan’s economy was unable to double its real per capita income. In contrast, its carbon emissions per capita more than doubled because in the 1980s, its economic growth averaged more than 6%, while in the 1990s, its economic growth was below 4%. Similarly, economic growth was approximately 4% from 2000 to 2010 and was less than 2% in 2001 and 2010. Likewise, economic growth was approximately 4% from 2011 to 2018 [4]. This means that Pakistan’s economic growth fluctuated between 4% and 5% during the study period. The government has to ensure high sustainable growth to produce an increase in per capita income, as this study finds that a further increase in income will help Pakistan mitigate environmental degradation. High and sustainable economic growth will only be possible if there is consistency in policies and, above all, if there is political stability, which may ensure consistency in policies. Another reason for the slow increase in real per capita income is energy shortages and the energy crisis that has worsened since the early 2000s. Thus, a sound and feasible energy policy is required to address the energy crisis on the one hand and to ensure a sustainable environment and development on the other hand. The government is advised to speed up hydro projects and develop energy projects with renewable resources to address energy shortages in Pakistan and make it possible to provide environmentally friendly energy. In contrast, the government is interested in alleviating the energy crisis and not taking into consideration the environmental issues associated with coal projects that are underway. By 2022, the indigenous and exogenous cumulative coal capacity will reach 4290 MW and 5201 MW, respectively. These coal projects will put more pressure on environmental quality in Pakistan. Currently, Pakistan is producing just 431 MW from solar energy, and very little has been done to explore the wind energy potential. The results of this study indicated that export variety does not worsen environmental degradation; thus, firms in Pakistani industries have to be encouraged to import environmentally friendly (green) technology in the form of minimum possible tariffs. Likewise, incentives have to be provided to encourage research and development in the industrial sector so that firms may become more efficient not only in production but also in energy use. In addition, the government has to offer special incentives in the form of tax relaxation/exemption to industries to adopt the green concept. This will not only enable exporters to enhance export variety but also help Pakistan curb environmental degradation. The government of Pakistan is required to generate a safe environment for FDI, as it would lead to better economic performance and lessen environmental degradation. Similarly, the government has to encourage the private sector and FDI in the energy sector along with special incentives (for instance, tax exemptions or relaxing taxes and lessening the cost of investment by facilitation services) to produce energy from renewable resources.
